# Acupuncture in acute herpes zoster pain therapy (ACUZoster) – design and protocol of a randomised controlled trial

**DOI:** 10.1186/1472-6882-9-31

**Published:** 2009-08-12

**Authors:** Johannes Fleckenstein, Sybille Kramer, Philipp Hoffrogge, Sarah Thoma, Philip M Lang, Lukas Lehmeyer, Gabriel M Schober, Florian Pfab, Johannes Ring, Peter Weisenseel, Klaus J Schotten, Ulrich Mansmann, Dominik Irnich

**Affiliations:** 1 Multidisciplinary Pain Centre, Department of Anaesthesiology, University of Munich, Munich, Germany; 2 Department of Dermatology and Allergy Biederstein, Technische Universität München, Munich, Germany; 3 Division of Environmental Dermatology and Allergy, Helmholtz Zentrum München, Germany; 4 TUM, ZAUM-Center for Allergy and Environment, Munich, Germany; 5 Department of Dermatology and Allergology, Ludwig-Maximilians-University, Munich, Germany; 6 Institute of Medical Information Technology, Biometry and Epidemiology, University of Munich, Germany

## Abstract

**Background:**

Acute herpes zoster is a prevalent condition. One of its major symptoms is pain, which can highly influence patient's quality of life. Pain therapy is limited. Acupuncture is supposed to soften neuropathic pain conditions and might therefore act as a therapeutic alternative. Objective of the present study is to investigate whether a 4 week semi-standardised acupuncture is non-inferior to sham laser acupuncture and the anticonvulsive drug gabapentine in the treatment of pain associated with herpes zoster.

**Methods/Design:**

Three-armed, randomised, placebo-controlled trial with a total follow-up time of 6 months. Up to estimated 336 patients (interim analyses) with acute herpes zoster pain (VAS > 30 mm) will be randomised to one of three groups (a) semi-standardised acupuncture (168 patients); (b) gabapentine with individualised dosage between 900–3600 mg/d (84 patients); (c) sham laser acupuncture. Intervention takes place over 4 weeks, all patients will receive analgesic therapy (non-opioid analgesics: metamizol or paracetamol and opioids: tramadol or morphine). Therapy phase includes 4 weeks in which group (a) and (c) consist of 12 sessions per patient, (b) visits depend on patients needs. Main outcome measure is to assess the alteration of pain intensity before and 1 week after treatment sessions (visual analogue scale VAS 0–100 mm). Secondary outcome measure are: alteration of pain intensity and frequency of pain attacks; alteration of different aspects of pain evaluated by standardised pain questionnaires (NPI, PDI, SES); effects on quality of life (SF 36); analgesic demand; alteration of sensoric perception by systematic quantitative sensory testing (QST); incidence of postherpetic neuralgia; side effects and cost effectiveness. Credibility of treatments will be assessed.

**Discussion:**

This study is the first large-scale randomised placebo controlled trial to evaluate the efficacy of acupuncture compared to gabapentine and sham treatment and will provide valuable new information about the clinical and physiological effects of acupuncture and gabapentine in the treatment of acute herpes zoster pain. The study has been pragmatically designed to ensure that the study findings can be implemented into clinical practice if acupuncture can be shown to be an effective treatment strategy in acute herpes zoster pain.

**Trial registration:**

NCT00885586

## Background

Herpes zoster is a distinctive syndrome caused by reactivation of varicella zoster virus (VZV). It is characterized by a painful, blistering skin eruption following dermatomal distribution. As cellular immunity to VZV decreases with age, stress or because of immunosuppression, the virus reactivates and travels along the sensory nerves to the skin, causing distinctive prodromal pain followed by eruption of rash [1]. The diagnosis of herpes zoster is clinically based on the characteristic appearance of the rash. Only in clinically unclear cases lesional detection of VZV-DNA by PCR might be used to confirm the diagnosis.

Herpes zoster can occur at any age but most commonly affects the elderly population. It is estimated that approximately 1 in 3 people will develop herpes zoster during their lifetime. The incidence of herpes zoster and the rate of herpes zoster-associated complications increase with age [2,3].

Symptoms that herald herpes zoster include pruritus, dysesthesia and pain along the distribution of the involved dermatome. The most distressing symptom is typically pain and the most feared complication beside CNS or ocular involvement is postherpetic neuralgia (PHN), the persistence of pain after rash healing. PHN is defined as pain persisting more than 3 months after the rash has resolved [4-6]. In patients aged over 70 years almost half develop PHN after acute herpes zoster [7]. Both, the acute pain associated with herpes zoster and the chronic pain of PHN, have multiple adverse effects on health-related quality of life. Different types of pain and other sensory symptoms are found in patients with herpes zoster, and these vary greatly with respect to their presence, location, duration, intensity, and quality [8]. These pain conditions cause substantial interference with physical, emotional, and social functioning [5,8] and result in increased health care costs [9].

The development of effective strategies for the prevention and treatment of pain associated with herpes zoster and PHN is therefore an unmet public health need [8]. Early recognition and treatment of herpes zoster can reduce acute symptoms and may also reduce the occurrence of PHN [9].

The evidence base supports the oral use of tricyclic antidepressants, certain opioids, and gabapentinoids to prevent or treat PHN [10]. Authors in this meta-analysis were able to extract an appreciable frequency of minor adverse events. The most frequently reported adverse events are dizziness and sedation, thus decreasing patients' daily quality of life and compliance [10]. In addition, current opinion is that existing interventions do not completely prevent or adequately treat all cases of herpes zoster pain and PHN [10-12].

Acupuncture might figure as an alternate. There is some evidence, that acupuncture might be favourable in the treatment of neuropathic pain conditions [13]. In addition acupuncture is known to be a safe treatment poor in adverse effects [14]. Though, there are only some smaller studies about acupuncture treatment in patients with herpes zoster [15-19] showing controversial results. Broad clinical studies are missing.

### Aim of the study

The primary objective of the trial presented is to investigate whether a 4 week semi-standardised acupuncture is non-inferior to the anticonvulsive drug gabapentine and sham laser acupuncture as placebo control in the treatment of pain associated with herpes zoster in addition to standardised analgesics. Secondary objectives include alteration of pain intensity and frequency of pain attacks, alteration of different aspects of pain evaluated by standardised pain questionnaires (NPI, PDI, SES), effects on quality of life (SF 36), analgesic demand, alteration of sensoric perception, incidence of postherpetic neuralgia, side effects and cost effectiveness.

## Methods/Design

### Design

**Figure 1:**
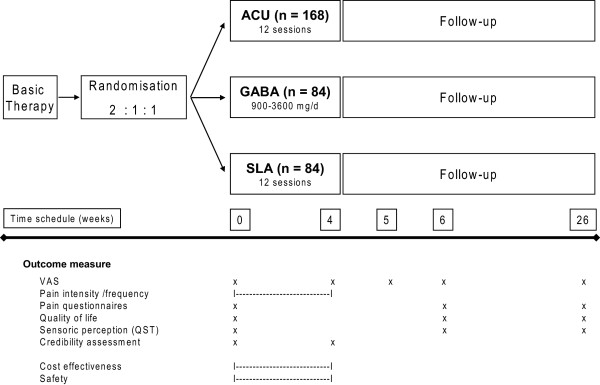
**Trial design, time schedule, and outcome parameters of the ACUZoster study**. Acupuncture (ACU), Gabapentine (GABA), Sham Laser Acupuncture (SLA). Outcome measures: Main outcome measure: Alteration of Pain intensity (VAS); Secondary outcome measure: alteration of pain intensity/frequency (evaluated by diary); pain questionnaires (including: NPI, PDI, SES, please refer to Table 3); quality of life (SF-36) Sensoric perception evaluated through qualitative sensory testing (QST); credibility assessment according to Vincent; cost effectiveness and safety aspects.

The ACUZoster study is a three-armed, partially blinded randomised placebo-controlled trial investigating the efficacy of (a) acupuncture versus (b) gabapentine or (c) sham laser acupuncture in the treatment of pain associated with acute herpes zoster in addition to a standardised analgesic regimen respectively (Figure 1). Patients are blinded regarding acupuncture and sham laser acupuncture treatment. After randomisation, patients in the acupuncture and sham laser acupuncture group receive 12 treatment sessions over a period of 4 weeks. In the gabapentine group, all patients receive the drug-uptake according to a standardised scheme. Appointments with gabapentine group patients depend on their needs and might happen up to 12 times in 4 weeks, but at least once a week. All patients are treated with standard antiviral, local and basic analgesic regimen. Furthermore patients can receive analgesic escape medication, so that a sufficient pain therapy is guaranteed. The total follow-up study period per patient is 6 months. Ethical approval has been given by the Ethics Committee of the University of Munich, Munich, Germany (registration 159-08).

### Patients

The ACUZoster trial aims to recruit up to a maximum of 336 patients. Recruitment for the trial started in November 2008. For inclusion patients must meet the following criteria: confirmed diagnosis of acute herpes zoster (recruitment at the Departments of Dermatology and Allergology, Ludwig Maximilians Universität and Technische Universität München, Munich, Germany), pain intensity > 30 mm on a visual analogue scale (VAS 0–100 mm), standardised antiviral therapy with either brivudin (125 mg/d p.o.) or aciclovir (3 × 5–10 mg/kg/d KG i.v. or 5 × 800 mg/d p.o.).

Main exclusion criteria are: Patients with insulin-dependent diabetes mellitus or other diseases influencing the peripheral sensibility (e.g. polyneuropathia, chronic pain syndromes, cutaneous irritations i.e. burns); patients under age (< 18 years); non-compliance; pregnancy or lactation; surgery within the last 3 month; severe heart/lung/kidney disease; diseases influencing the quality of life; psychiatric diseases (e.g. depression, schizophrenia, dementia); chronic intake of analgesics, neuroleptics, antidepressants, corticoids, alpha-agonists; acupuncture, transdermal electric neurostimulation or other Complementary and Alternative Medicine treatment within the last 4 weeks; contraindications according to the summary of product information against analgesic treatment (i.e. metamizol, paracetamol, tramadol, morphine) or the investigational medicinal products (gabapentine, acupuncture needles).

### Participating physicians

Participating trial physicians form part of the Multidisciplinary Pain Centre, Department of Anaesthesiology, University of Munich, Germany. All are experienced in pain treatment. Their acupuncture training reaches at least the level of "A-diploma" from one of the major German acupuncture societies (140 hours of curricular teaching) such as more than two years practical skills training at the Multidisciplinary Pain Centre. All physicians are experienced in working in clinical trials.

### Randomized treatment allocation, and sample-size estimation

The randomization to one of the three arms of the study has been carried out by the Institute of Medical Information Technology, Biometry and Epidemiology, University of Munich, Germany (IBE). The investigators established a computer-based randomisation procedure (Randoulette^®^) which after including the patient to the trial allocates subjects to the respective arms balancing their age and gender

We conduct a 3-armed study, which shall prove the non-inferiority of an experimental therapy (acupuncture) against a reference therapy (gabapentine) by simultaneous placebo control (sham laser acupuncture). The sample size estimation was based on the approach of Pigeot, Schäfer, and Röhmel [20]. Following assumptions were made on the basis of a prior study [13].

• VAS change under acupuncture treatment: μ_E _= 15 mm

• VAS change under gabapentine (reference treatment): μ_R _= 15 mm

• VAS change under sham treatment: μ_P _= 5 mm

• Noninferiority margin: θ = 50% of the difference between the reference and the sham treatment (i.e. 5 mm VAS)

• σ = 15,6 (estimated standard deviation within groups)

• α = 0,025 (one-sided)

• β = 0,2 (power: 80%)

The necessary sample size under these assumptions was estimated as n = 336, with an optimal allocation of 168 patients to the acupuncture group and 84 patients each to the reference and placebo group. The dropout rate should not be higher than 10%.

To allow for early termination, the study was designed to be assessed in four equally spaced interim analyses and one final analysis. Therefore, α-levels were adjusted according to O'Brien-Fleming to maintain the assumed 0,025-level for the overall study analysis [21].

Interim analysis 1 (20% of all patients): 0,00000

Interim analysis 2 (40% of all patients): 0,00039

Interim analysis 3 (60% of all patients): 0,00368

Interim analysis 4 (90% of all patients): 0,01102

Overall study analysis (100% of all patients): 0,02113

### Statistical analysis

Establishing noninferiority of the experimental therapy E (acupuncture) to the reference therapy R (Gabapentin) requires hierarchical testing of the following hypotheses using t tests [20]:



In a first step,  is tested using the appropriate alpha level. Only if  is rejected, the second hypothesis *H*_0 _is tested, using the same alpha level. If *H*_0 _is rejected, noninferiority is established. If the first step does not lead to the rejection of , noninferiority cannot be established at this stage of the study.

### Interventions

#### Basic therapy (all groups)

All patients are treated by the attending dermatologists immediately after diagnosis of herpes zoster with a standardised antiviral therapy with either brivudin (125 mg/d p.o.) or aciclovir (3 × 5–10 mg/kg/d KG i.v. or 5 × 800 mg/d p.o.) as well as a symptomatic therapy of rash with local antiseptics according to the guidelines of the German Dermatological Society (Deutsche Dermatologische Gesellschaft DDG, [22]).

In addition all patients have the possibility to receive a standardised analgesic treatment, according to WHO recommendations: step 1: nonopioid analgesics (Metamizol 4 × 1.0 g p.o. (4 × 40 gtt). Paracetamol 4 × 1.0 g), step 2: additionally moderate opioids (tramadol, maximum dose 600 mg/d), step 3 recommends use of stronger opioids (morphine). Escape medication available for step 2 and 3 are tramadol respectively morphine drops. Patients are not allowed to use other analgesics or analgetic therapies.

#### Acupuncture treatments (a + c)

Patients in the acupuncture group and the sham laser group will receive an acupuncture treatment. Acupuncture treatment is semi-standardised. Obligatory basic points with and without electrical stimulation have to be chosen. In addition facultative individual points can be chosen according to the diagnostic pattern and its corresponding meridian systems (Table 1). Detailed diagnosis according to Western and Chinese diagnostic considerations (including pulse and tongue diagnosis) is assessed. Chosen acupuncture points are recorded.

**Table 1:** Acupuncture Treatment protocol

AcuPoint	Traditional Name	Indication (acc. to TCM)
**Obligatory basic points applied with electrical stimulation**		

LI 4	He Gu	pain; skin diseases; restlessness; inflammation; head affections

LI 11	Qu Chi	strengthen the immune system; pain; skin diseases; restlessness; inflammations

dependent on the primary diagnostic pattern **either**		

LR 3	Tai Chong	headache; psychosomatic symptoms with stress and restlessness; muscular tension; spasmolytic

GB 34	Yang Ling Quan	pain and tension in muscles and tendons

**or**		

SP 10	Xue Hai	immunmodulatoric effect; skin diseases

BL 40	Wei Zhong	skin diseases; local burning and heat symptoms

**Obligatory basic points (electrical stimulation optional)**		

Points on the meridian system corresponding to the primary diagnostic pattern (at least 2)		

Segmental points (standard segmental points e.g. Huatuo or BL points, as well as Ah Shi points, at least 2)		

Local points (at least 4)		

**Facultative points can be chosen according to diagnosis and/or symptoms**		

Standard acupuncture points, Ah Shi points, Microsystempoints (e.g. ear), myofascial triggerpoints		

The treatment strategies for acupuncture were developed based on a consensus process with experienced acupuncture experts representing members of the faculty of one of the major German societies for medical acupuncture: German Medical Acupuncture Association (*Deutsche Ärztegesellschaft für Akupunktur*, DÄGfA).

Both treatment groups (needle and sham laser) consist of 12 sessions within 4 weeks (3 sessions/week). Each session is supposed to last 20 minutes. Resting time after both treatments is 20 minutes.

(1) The needle acupuncture technique (a) used in this trial is performed using expendable needles (Seirin^® ^0.15 × 20 mm or 0.3 × 30 mm and Asiamed^® ^0.25 × 40 mm) at defined acupuncture points and treatment areas. After needle insertion, the needle is manipulated until the subject obtains the deqi response (a deep aching or full feeling at the needle). Needle techniques include very point technique and dry needling. Further manipulation can be obtained applying an electro acupuncture device (AS Super 4 Han, schwa-medico, Ehringshausen, Germany).

(2). Sham laser acupuncture (c) is applied at equivalent points as needle acupuncture, approaching a non-functioning laser pen which had been deactivated before by the manufacturer (Handy CW 100, schwa-medico). Only red light is emitted. For emphasis of the imaginary power of this sham procedure, visual and acoustic signals are accompanying the red light emission. Patients are treated 45 sec. without skin contact.

#### Gabapentine (b)

Patients are treated individually with gabapentine 900 mg/d – 3600 mg/d. According to the recommended scheme given by the manufacturer (Table 2), the initial dose of 300 mg/d is (1) gradually augmented up to a daily dose of 900 mg and can (2) be increased dependent on the patients needs (maximum dose: 3600 mg/d). When symptoms release for at least one week, reduction of daily gabapentine amount is aspired.

**Table 2:** Gabapentin Scheme

**Day Time**	**8°° a.m**.	**14°° a.m**.	**22°° p.m**.
Day 1	-	-	300 mg

Day 2	300 mg	-	300 mg

Day 3	300 mg	300 mg	300 mg

*Increase depends on patients needs*			

Day n	300 mg	300 mg	600 mg

Day n + 1	600 mg	300 mg	600 mg

Day n + 2	600 mg	600 mg	600 mg

...			

Maximum dose	1200 mg	1200 mg	1200 mg

Table 2 is demonstrating the gabapentine intake scheme to reach the wanted therapeutic dosage. n is in sequence of the days since start of the therapy, at least n = 4.

Patients are scheduled for re-assessment at least once a week.

### Ethics

The trial has been approved by the Ethics Committee of the University of Munich such as the national component authority (Bundesinstitut für Arzneimittel und Medizinprodukte BfArM). The study protocol is in accordance to the declaration of Helsinki and the „ICH E6 Guideline for Good Clinical Practice”. Written informed consent is obtained from all patients.

### Monitoring

The study will be conducted according to common guidelines for clinical trials (Declaration of Helsinki ICH-GCP, Version Seoul 2008, cf. ), including certification by an external audit according to ICH-GCP at the Institute for Medical Informatics, Biometrics and Epidemiology (IBE), Ludwig-Maximilians-University, Munich, Germany. Data protection such as adequate quality and safety control according to the guidelines for good clinical practice (CPMP/ICH/135/95) is assured. Trial registration is EudraCT 2006-004698-86.

### Blinding

Patients are blinded and told they would receive either acupuncture or laser acupuncture or a medical treatment. They are told that one treatment only could be less effective than a series of applications. Instead of true laser acupuncture, a sham laser acupuncture treatment is performed. In addition, examiners providing the sensory testing and that are involved with the data management are blinded about patients' treatment.

### Outcome measures

#### Main outcome measure

Alteration of pain intensity before and 1 week after treatment sessions (visual analogue scale VAS 0–100 mm, with 0 being no pain and 100 being the maximum imaginable pain.

#### Secondary outcome measure

Alteration of pain intensity and frequency of pain attacks, analgesic demand, evaluated by diary; alteration of different aspects of pain evaluated by standardised pain questionnaires (NPI, PDI, SES); effects on quality of life (SF 36), alteration of sensoric perception by systematic quantitative sensory testing (QST); incidence of postherpetic neuralgia; side effects and cost effectiveness. QST measurements are taken before and 1 week and 6 months after treatment. For detailed information please refer to Table 3.

**Table 3:** Secondary outcomes

→ Variation of the primary outcome (averaged pain intensity)
→ Frequency of pain attacks per day

→ Pain intensity:

▪ Pain at rest

▪ Pain attack

→ Time to symptom alleviation

→ Analgesic demand

→ Side effects of the therapy

→ Standardised pain questionnaires

▪ Neuropathic Pain questionnaire (NPI)

▪ Quality of life (SF 36)

▪ Pain Disability Index (PDI)

▪ Pain description acc. to Geissner (SES)

▪ Pain discomfort list acc. to van Zerssen

→ Quantitative sensory testing (QST):

▪ thermic sensation:

▪ cold and warm detection threshold (CDT + WDT)

▪ thermic difference threshold (TSL)

▪ cold and heat pain threshold (CPT + HPT)

▪ tactile detection threshold

▪ mechanical pain threshold

▪ mechanical pain intensity

▪ mechanical allodynia

▪ wind up phenomenon

▪ vibration threshold

▪ pressure pain threshold

→ Change of rash before and after treatment

→ Incidence of postherpetic neuralgia 6 month after treatment

→ Credibility

→ Cost effectiveness

→ Side effects

### Credibility assessment

Expectations about outcome are the main modifying variables of the placebo effect according to Strauss-Blasche [23]. Patients are therefore asked to evaluate whether their satisfaction and expectations are met through a specific questionnaire according to Vincent which is comprised of 4 items: (1) How confident do you feel that this treatment can alleviate your complaint?; (2) How confident would you be in recommending this treatment to a friend who suffered from similar complaints?; (3) How logical does this treatment seem to you?; (4) How successful do you think this treatment would be in alleviating other complaints? [24]. Patient satisfaction is measured with a 10 point visual analogue scale (VAS) at the end-point of the study.

### Data entry

Data entry is done with SPSS statistical software system (SPSS Inc., Chicago, IL; version 15.0). Data analysis will be done with SAS/STAT^® ^Software (SAS Institute Inc., Cary, NC, USA) All data entry will be carried out twice.

## Discussion

To our knowledge, the ACUZoster study is the first clinical study to investigate the effectiveness of an acupuncture treatment for acute herpes zoster pain in direct comparison to a standard analgesic treatment with gabapentine and to a sham laser acupuncture treatment in a three-armed, randomised controlled clinical trial. Compared to previous studies of acupuncture in the treatment of herpes zoster, this study has a more rigorous methodology and will include more patients.

As pain in Herpes zoster is a prevalent symptom, zoster neuralgia figures an established model to investigate neuropathic pain therapies [10]. Therefore we believe that the expected results will not only confirm the present basic analgesic therapy in zoster neuralgia, it will also be possible to reason by analogy on other painful neuropathic conditions. However, depending on different age and gender distribution, thus leading to different pain perceptions within the patients' cohort, we can not control each patient's symptoms individually. We think that the balanced randomisation process accounts on this source of bias.

Inclusion and exclusion criteria were hold pragmatic in order to facilitate screening and recruitment. Exclusion criteria (besides standard items such as pregnancy or contra indications to the study medication) are diseases interfering with the patients' sensory perception. Our inclusion and exclusion criteria are based on further trials evaluating sensations and on recommendations found in the literature [11,25,26].

### Acupuncture versus gabapentine

No single treatment has been shown to be completely effective for all sufferers of acute herpes zoster pain, and in the practical clinical scenario combinations of analgesic drugs are usually required to achieve partial relief of pain. Although there is an increasingly large number of trials that compare various analgesics to placebo, very few directly compare single therapies for which an evidence base exists, or address the issue of combining treatments [27]. Current evidence is based on a small number of clinical trials supporting the oral use of tricyclic antidepressants, certain opioids, and gabapentinoids in PHN [10]. The pooled results for e.g. gabapentine give a number needed to treat (50% pain reduction) of 4.39. This efficacy is independent of the maximum applied dosage [10]. Gabapentine is one of the most commonly used analgesics in the treatment of neuropathic pain. Despite, adverse effects are reported quite often. Patients suffer especially from dizziness and sedation. The number needed to harm for gabapentin is 4.07 for minor harm and 12.25 for major harm [10]. That means that the number required to treat is same as the number needed to provoke adverse events.

Even as the use of acupuncture has been reported being promising by smaller trials in neuralgia [28], neuropathic pain [13,29] or postherpetic conditions [15], all these trials come not up to enough evidence to recommend acupuncture as standard regimen.

To our knowledge, this is the first study evaluating the effectiveness of acupuncture in a large scaled trial proofing its non-inferiority in comparison to the commonly used analgesic gabapentine, both in comparison to sham treatment.

### Control Procedure

There is a controversial discussion on the subject of control procedures for clinical acupuncture trials [24,30,31]. The use of superficial, or of deep, needle insertions at locations distant from real acupuncture points, also known as minimal or sham acupuncture control, is not suitable to constitute an inert placebo, as diverse non-specific physiological effects of needle stimulation have been observed. This procedure is more likely to merely evaluate needling effects regarding the depth or the site of needle insertion. There are various physiological antinociceptive effects which are inevitable when using needle insertion or related techniques. Development of so called placebo needles was an essential step in the methodology of research on mechanisms of acupuncture [31]. However, applying placebo needles in clinical trials implies methodological problems e.g. artificial setting, loss of credibility in acupuncture experienced by patients while mechano-sensitive Aβ-fibres are used, which by itself can activate pain inhibitory systems [32,33]. In addition, all of these methods have a common problem of compromising the therapist blinding.

In contrast, the choice of sham laser avoids activation of non-specific physiological effects such as stimulation of Aβ-fibres or C-fibres. Blinding of patients is easily achieved. Blinding both therapist and patients has been performed successful in a precedent trial [34], but was not applicable in this trial. The treatment setting of laser acupuncture carries equal weight to needle acupuncture in many respects e.g. attention, relaxation, and concentration on body sites distant from the affected painful area. All of these are potential factors which play an important role to both physicians and patients [35]. Using sham laser allows evaluation of the efficacy of analogous needling.

A limitation is that the therapist is still familiar with the difference of needle versus laser. In addition, technical devices are not directly comparable to the manual skill of acupuncture, and they are also liable to evoke different patient belief in treatment effects [36].

Given that the resolution to this controversy remains in doubt, the use of the sham laser methodology in this randomised single blinded study design avoids these mentioned concerns while still providing a valid control. Assessment of expectations is therefore still needed to demonstrate the level of belief in the treatments supporting our contention [23,37].

We chose sham laser acupuncture as a control as this is suggested to provoke only unspecific treatment effects [37]. For the blinded patients, the credibility of this sham procedure is enhanced by visual and acoustic signals accompanying the red light emission, imitating a working laser pen. The credibility assessment of the acupuncture and sham treatment will be rated. This is in agreement with studies prior to this investigation demonstrating very similarly belief for both therapies [38,39]; we claim that blinding of patients is effective and that the choice of the sham laser acupuncture as a control procedure is adequate. Patients are blinded as them is told that they will receive either acupuncture or a laseracupuncture treatment. It is mentioned that one treatment can be less effective and that sham treatments can be included. This way we try to consider the ethical responsibility towards the patient. The Ethics Committee at the University of Munich, Germany approved this approach being suitable.

## Conclusion

This study is the first large-scale randomised placebo-controlled trial to evaluate the efficacy of acupuncture compared to gabapentine and sham treatment. It can be expected to provide valuable new information about the clinical and physiological effects of acupuncture and gabapentine in the treatment of acute herpes zoster pain. The study has been designed pragmatically to ensure that its findings can be implemented into clinical practice if acupuncture or gabapentine are found to be effective treatment strategies in acute herpes zoster pain.

## Competing interests

The authors declare that they have no competing interests.

## Authors' contributions

JF helped conceiving of the study and drafted the manuscript. SK, PL, LL and GMS participated in the design of the study. Parts of the study constitute the topics of the medical thesis of PH and ST. FP, JR and JP participated in the design of the study and coordinate the study in their departments. AC performed the sample size estimation. KJS organises internal and external monitoring. UM conceived of the biometrical study design. DI conceived of the study, he participated in the design and coordination of the study and supervised drafting the manuscript. All authors read, and approved of the final manuscript.

## Pre-publication history

The pre-publication history for this paper can be accessed here:


